# The Effects of Beetroot Juice on Blood Pressure, Microvascular Function and Large-Vessel Endothelial Function: A Randomized, Double-Blind, Placebo-Controlled Pilot Study in Healthy Older Adults

**DOI:** 10.3390/nu11081792

**Published:** 2019-08-02

**Authors:** Tomos Jones, Emily Louise Dunn, Jamie Hugo Macdonald, Hans-Peter Kubis, Nicholas McMahon, Aamer Sandoo

**Affiliations:** 1 School of Sport, Health and Exercise Sciences, Bangor University, George Building, Bangor, Gwynedd, Wales LL57 2PZ, UK; 2 School of Human Movement and Nutrition Sciences, University of Queensland, St. Lucia, QLD 4067, Australia

**Keywords:** dietary nitrate, endothelial dysfunction, endothelial function, large-vessels, microvessels, blood pressure

## Abstract

Dietary nitrate (NO_3_^−^) has been reported to improve endothelial function (EF) and blood pressure (BP). However, most studies only assess large-vessel EF with little research on the microvasculature. Thus, the aim of the present pilot study is to examine NO_3_^−^ supplementation on microvascular and large-vessel EF and BP. Twenty older adults (63 ± 6 years) were randomized to a beetroot juice (BRJ) or placebo (PLA) group for 28 (±7) days and attended three laboratory visitations. Across visitations, blood pressure, microvascular function and large-vessel EF were assessed by laser Doppler imaging (LDI) with iontophoresis of vasoactive substances and flow-mediated dilatation (FMD), respectively. Plasma NO_3_^−^concentrations, BP and the presence of NO_3_^−^ reducing bacteria were also assessed. Plasma NO_3_^−^ increased following two weeks of BRJ supplementation (*p* = 0.04) along with a concomitant decrease in systolic and diastolic BP of approximately −6 mmHg and −4 mmHg, respectively (*p* = 0.04; *p* = 0.01, respectively). BP remained unchanged in the PLA group. There were no significant differences in endothelium-dependent or endothelium-independent microvascular responses between groups. FMD increased by 1.5% following two weeks of BRJ (*p* = 0.04), with only a minimal (0.1%) change for the PLA group. In conclusion, this pilot study demonstrated that medium-term BRJ ingestion potentially improves SBP, DBP and large-vessel EF in healthy older adults. The improvements observed in the present study are likely to be greater in populations presenting with endothelial dysfunction. Thus, further prospective studies are warranted in individuals at greater risk for cardiovascular disease.

## 1. Introduction

The endothelium is the inner most lining of the vasculature and plays a pivotal role in regulating vascular tone via the release of several vasoactive factors [1]. One such factor is nitric oxide (NO), which is a potent vasodilator that is important for maintaining vascular homeostasis [2]. Endothelial dysfunction (ED) is characterized by a reduction in NO bioavailability and can increase cardiovascular disease (CVD) risk, particularly in older individuals [3,4]. 

Vegetables such as beetroot and spinach contain high levels of nitrate (NO_3_^−^) and when consumed, they can increase NO bioavailability independently of the endothelium [5]. When ingested, dietary NO_3_^−^ is partially absorbed into the circulation and is later taken up by the salivary glands. Within the oral cavity NO_3_^−^ becomes 10- to 20-fold more concentrated in the saliva [6]. Further, salivary NO_3_^−^ is reduced to nitrite (NO_2_^−^) by commensal facultative anaerobic bacteria, located on the posterior region of the tongue. After swallowing, most of the salivary NO_2_^−^ is converted to NO in the acidic conditions of the stomach; however, a small percentage is transported into the circulation. Once in the circulation, NO_2_^−^ is converted to NO through enzymatic and non-enzymatic reactions [7,8,9,10,11]. This endothelium-independent production of NO is commonly described as the entero-salivary NO_3_^−^–NO_2_^−^–NO pathway [12]. A growing body of literature identifies dietary NO_3_^−^ as an exogenous source of NO, which has the capacity to improve endothelial function (EF) in individuals at risk of CVD [13]. 

A prospective study (over 14 years) in a cohort of older adults, reported that a diet low in NO_3_^−^ rich vegetables was correlated with the prevalence of CVD mortality [14], this conclusion is also in line with previous findings from the same research group [15]. Furthermore, the high NO_3_^−^ content of a Mediterranean and Japanese diet has been suggested to be associated with significant blood pressure (BP) improvements [16,17]. Diets, which are naturally rich in NO_3_^−^, supplements such as beetroot juice (BRJ), have also been demonstrated to have beneficial effects on BP [9,18,19,20,21,22,23,24,25]. Although these discoveries support the cardioprotective features of dietary NO_3_^−^, little is known about the underlying mechanisms. In humans, EF is commonly assessed in conduit arteries using flow-mediated dilatation (FMD), which measures the endothelium-dependent dilatory response to reactive hyperaemia. In a large cohort of 68 hypertensive patients, FMD responses were significantly improved following four weeks of daily BRJ consumption [19]. Furthermore, both systolic BP (SBP) and diastolic BP (DBP) were reduced following one week of the intervention and were sustained throughout the four-week supplementation period. Interestingly, there is limited research available that has investigated the effect of NO_3_^−^ supplementation on microvascular EF, despite the microvasculature being the primary regulator of systemic vascular resistance (SVR) and encompassing a larger surface area than the large vessels [26]. Moreover, microvascular ED is believed to precede ED in the larger blood vessels [27]. However, due to the heterogeneity of the endothelium, no clear associations can be made between vascular beds [28,29]. Therefore, it is important to understand the underlying principles of microvascular EF.

Gilchrist et al. examined the effects of a two-week BRJ intervention on microvascular function, large-vessel function and BP in individuals diagnosed with type II diabetes (T2D) [30]. Large-vessel function was assessed using FMD and nitroglycerine-mediated dilatation (endothelium-independent), while microvascular function was measured using laser Doppler imaging (LDI) with iontophoresis of endothelium-dependent (acetylcholine (ACh)) and endothelium-independent (sodium nitroprusside (SNP)) agonists [31]. Contrary to Gilchrist and colleagues’ hypothesis, the intervention did not result in any significant improvements in vascular function or BP. The authors concluded that subjects had irreversibly diminished vascular reactivity, due to having multiple risk factors for CVD. Nonetheless, a longer intervention of six weeks has been shown to improve FMD and reduce SBP in hypercholesterolemic patients, although no microvascular assessment was performed in this study [18].

Therefore, the present pilot study aims to examine the effect of BRJ supplementation on microvascular function, large-vessel EF and BP in older adults over a four-week period. We hypothesized that four weeks of daily supplementation with NO_3_^−^ rich BRJ will increase NO bioavailability, along with concomitant improvements in vascular function and BP.

## 2. Materials and Methods

### 2.1. Participants

**Figure 1:**
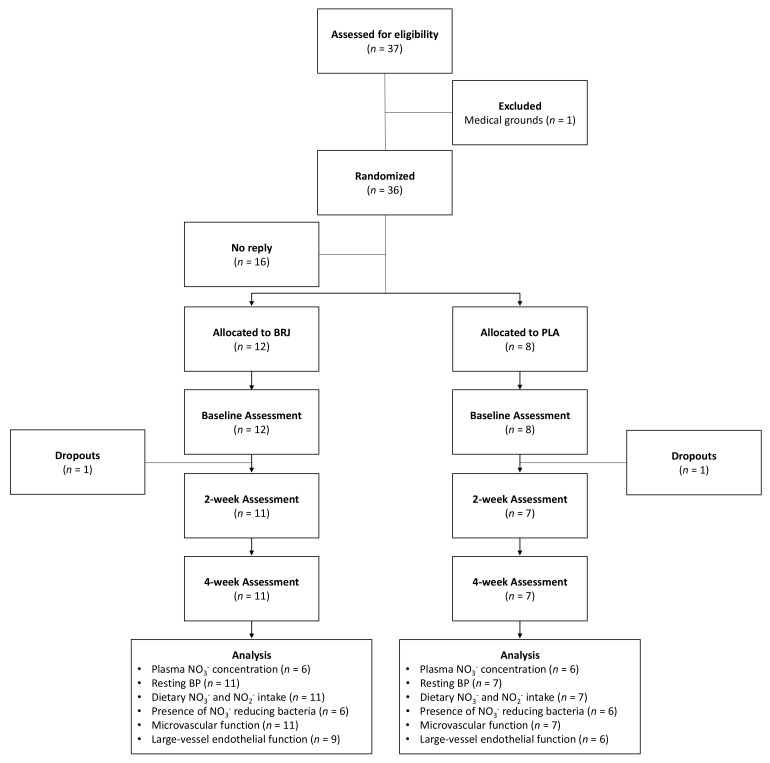
Consolidated standards of reporting trials (CONSORT) flowchart of study. BP = blood pressure; BRJ = beetroot juice; NO_3_^−^ = nitrate and PLA = placebo.

Thirty-seven healthy older adults were recruited and assessed for eligibility by e-mail, flyers and advertisements (Figure 1). Participants were allocated to each group using stratified randomization based on age (±3 years) and gender to ensure similarities between both arms. From the sample, 16 people did not reply to the invitation, one person was excluded on medical grounds. Therefore, 20 healthy older adults (age: 63 ± 6 years) participated in the present study. Due to personal reasons, two individuals withdrew from the study following a week of their intervention. Participants were briefed on the nature and the purpose of the investigation before written consent was taken along with a short demographic questionnaire to ensure that they satisfied the study criteria. 

To avoid the effects of the menstrual cycle, only men and post-menopausal women aged 55 or older were recruited for the study. Exclusion criteria included any history of acute coronary syndrome or established CVD, use of antihypertensive medication, chronic diseases or acute illnesses, highly trained/active individuals, diets rich in green leafy vegetables and habitual use of antibacterial mouthwash. All experimental procedures were performed in accordance with the ethical standards of the Declaration of Helsinki and approved by the institutional ethics committee (S02-16/17).

### 2.2. Experimental Design

A randomized placebo-controlled trial utilizing a parallel study design was used to test the hypothesis. Those assessing the outcomes were blinded to the conditions and participants were never told which treatment they were allocated to, only that both drinks had health benefits. For the duration of the study, all participants were required to attend three laboratory visits, separated by 14 (±7) days. All visits were performed in a thermoregulated laboratory (21–22 °C), which started between the hours of 07:30–09:00 am and lasted approximately 2 h. Participants arrived following a 12-h fast and were asked to refrain from caffeine. They were also instructed to avoid strenuous exercise 24 h prior to testing. Lastly, participants were asked to complete a three-day food diary and the seven-day international physical activity questionnaire (IPAQ) [32] before attending the laboratory sessions. Over the period of the study, all participants were asked to maintain their usual diet and physical activity levels.

During each visit, anthropometric data was collected prior to any other assessments, which included height, body mass, body mass index (BMI) and body fat percentage (TANITA C-300; Tanita Ltd, Japan). Venous blood samples were collected into ethylenediaminetetraacetic acid (EDTA) collection tubes and were centrifuged immediately. The plasma was separated into 500 µL aliquots before being transferred to a −80 °C freezer for later analysis. Following the blood sample, a tongue scrape sample, containing potential nitrate reducing bacteria, was collected from the posterior region of the tongue. Samples were transferred into a NO_3_^−^ medium and immediately incubated. Subsequently, participants were asked to lie down for 10 min before three separate automated BP measurements were taken (separated by 2 min) and the mean value was recorded. Whilst remaining in a recumbent position, vascular function of the small and large blood vessels was assessed (separated by 15 min). 

### 2.3. Drink Intervention

Daily consumption of the active or placebo (PLA) treatment was instructed for the duration of their enrolment. The active treatment was a 70 mL NO_3_^−^ rich BRJ drink (Beet-It Sport Shot 400; James White Ltd, Ashbocking, UK), providing approximately 400 mg of NO_3_^−^ each day. For the PLA group, prune juice (Sunsweet Growers Inc., Kingston upon Hull, UK) was selected because of the negligible NO_3_^−^ levels (<0.01 mM) [33] and similar consistency and colour to BRJ. Additionally, carbohydrate and fibre content of both treatments have been reported to be similar [33]. All participants were asked to consume their allocated drink in the morning and to keep it consistent for the duration of the study, with the exception of testing days. 

### 2.4. Dietary NO_3_^−^ and NO_2_^−^ Intake 

The NO_3_^−^ and NO_2_^−^ content was determined by a database containing NO_3_^−^ and NO_2_^−^ values from 7703 foods and beverage records from 432 publications [34]. To develop the most accurate and appropriate values in terms of the study population location, a similar method previously described [35] was used with priority given to: 1) The UK/European countries from 1990 to present; 2) the UK/European countries from 1960–1989; 3) countries with predominately Western diets (Australia, U.S and Canada) from 1990 to present; 4) countries with predominately Western diets from 1960–1989 and 5) countries with predominately non-Western diets. The cut-off dates were chosen to account for changes in laboratory methods, food preservation techniques and manufacturing technologies (the addition of ascorbate during meat processing, added to reduce the formation of nitrosamines), and legislation regulating the amounts of NO_3_^−^ and NO_2_^−^ used in the curing process created significant reductions in food and beverages [36,37]. 

NO_3_^−^ and NO_2_^−^ intake was calculated by multiplying the food or beverage item consumed (grams per day) as the weighted mean value of that item identified using the multistep process mentioned above. Weighted mean was selected as some mean values were adversely affected by extreme values. If a NO_3_^−^ or NO_2_^−^ value was unattainable a value of 0 mg/g was assigned to the food or beverage item. Cooking methods such as baked, blanched, boiled, broiled, cooked, fried, microwaved, raw and steamed were reported when relevant and available. 

If a food or beverage item’s serving amount was missing from the recall data, the recommended serving size amount was calculated using summary estimates (in grams) as defined by standard serving sizes in nutritional analysis software [38,39]. In the case of multicomponent foods (e.g., pizza, juices, salad mix, soup and curries), nutrient values were determined by calculating the NO_3_^−^ and NO_2_^−^ content of all ingredients contained in the recipe list, or in the case of commercial products, looking at the ingredients list or using the recipe reproduction [40]. Recipes were selected from reliable and common online kitchen resource materials. Total NO_3_^−^ and NO_2_^−^ (mg/day) were determined by calculating the sum of daily NO_3_^−^ and NO_2_^−^ values.

### 2.5. Presence of NO_3_^−^ Bacteria

A tongue scraping was collected from each participant during their first visit following an oral examination to ensure there were no signs of any mouth infections. The scrapings were performed using a disposable acrylic spatula from the posterior region of the tongue and were collected into sterile disposable pipette (Fisher Scientific, Cat. No. 13489108). Samples were transferred into 6 mL of NO_3_^−^ rich medium (Ingredients per litre of deionized water: Pancreatic Digest of Gelatin 5.0 g; Beef Extract: 3.0 g; Potassium Nitrate: 1.00 g; Hardy Diagnostics, Cat. No. K42) and incubated for 54 h at 37 °C, 95% humidity and 5% CO_2_. To ensure a similar inoculum size, the weight gain of the broth was kept constant at approximately 10 µg.

Following incubation, 1 mL of the NO_3_^−^ broth was extracted through a Puradisc 25 Polyethersulfone 0.2 µm sterilizing grade filter (Fisher Scientific, Cat. No. 6780-2502) to ensure that any bacteria were eliminated. Content of the syringe was then emptied into separate 500 µL aliquots and immediately frozen at –80 °C for later analysis of NO_3_^−^. The levels of NO_3_^−^ in the medium were analysed before and after incubation. Reductions in NO_3_^−^ levels are expressed as a percentage change (∆NO_3_^−^%), in order to determine the presence of NO_3_^−^ reducing bacteria. All the NO_3_^−^ analyses are described in more detail in Section 2.8. 

### 2.6. Microvascular Function

Iontophoresis administration of ACh (Miochol-E, Novartis, UK) and SNP (Nitroprussiat Fides, Rottapharm SL, Spain) was performed using an iontophoresis controller (MIC-Ie, Moor Instruments Ltd, UK) in order to assess cutaneous endothelium-dependent and endothelium-independent vasodilation, respectively. Perfusion changes on the anterior surface of the forearm in response to the delivery of both vasoactive drugs were assessed using LDI, which measures blood flux (AU). The full protocol that was used for this study has been described in detail previously [41]. In summary, one baseline scan was performed before a series of ten scans with an iontophoresis charge of 30 µA was delivered to administer 1% ACh and 1% SNP. ACh and SNP drugs were diluted with 0.9% saline and delivered into the skin via an anode and cathode internal electrode Perspex chamber (Ø22 mm; ION 6, Moor Instruments Ltd, UK), respectively. Following ten scans with iontophoresis, two further recovery scans were performed without the delivery of the vasoactive drugs. 

The exposure-time-response protocol took approximately 15–20 min and all of the scans were performed in natural lighting conditions, with most of the ambient lighting restricted. Additionally, the settings of the laser Doppler imager (moorLDI2-IR, Moor Instruments, Axminster, Devon, UK) were kept consistent for all scans. Measurements of perfusion were carried out offline using the moorLDI Review V6.1 software and results are presented as a percentage change in perfusion from baseline ($\frac{\mathrm{Peak}\;\mathrm{Flux}-\mathrm{Baseline}\;\mathrm{Flux}}{\mathrm{Baseline}\;\mathrm{Flux}}\times 100=\mathrm{ACh}\%\;\mathrm{or}\;\mathrm{SNP}\%$).

### 2.7. Large-Vessel Endothelial Function

The FMD procedure is a non-invasive technique that is commonly used on the brachial artery as an assessment of global large-vessel EF. Several published guidelines are available for this technique [42,43,44], however the present study used Sandoo and Kitas’ protocol [41]. Briefly, a 2 min baseline ultrasound scan of the brachial artery was followed by 5 min of arterial occlusion, achieved by inflating a BP cuff that was placed around the wrist to 220 mmHg. After a 5 min period of ischemia, the BP cuff was deflated rapidly, and a further 3 min scan of the artery was performed.

A Siemens Acuson X300 Ultrasound scanner was used with a multifrequency linear-array vascular probe set at 7.3 MHz (Siemens PLC, Camberley, UK) to perform the FMD procedure. B-mode images were captured at 15 frames per second to record a 120 s baseline and a 210 s clip following 5 min of occlusion. To capture the initial reactive hyperaemic response to the deflation of the BP cuff, the recording was initiated 30 s before cuff release; therefore, only 180 s was used for the analysis. Images were analysed offline using an automated edge detection software (Brachial Analyser, Medical Imaging Applications, USA). The Brachial Analyser software is capable of detecting the peak of the R-wave, therefore, this inbuilt feature was used to include only the images at the peak of the R-wave. Frames that did not meet the recommended quality standard (confidence threshold <70%) were rejected. From the frames that were accepted, the change in diameter from baseline to peak was calculated as follows, $\frac{\mathrm{Peak}\;\mathrm{Diameter}-\mathrm{Baseline}\;\mathrm{Diameter}}{\mathrm{Baseline}\;\mathrm{Diameter}}\times 100$ = FMD%. To account for the differences in baseline diameter, all the data was allometrically scaled as per the Atkinson and Batterham guidelines [45]. The coefficient of variation for the sonographer (DTJ) was reported as 8.5% 

### 2.8. Analysis of NO_3_^−^ Concentration

Whole venous blood samples were collected into EDTA collection tubes following a 12 h fast. Samples were immediately stored on ice or placed in a pre-chilled (4 °C) centrifuge. Once samples were centrifuged, 500 µL plasma aliquots were instantly stored at −80 °C. Prior to analysis, plasma samples were thawed and centrifuged again through an Amicon 30 kDa molecular weight filter to reduce protein content [46]. Similarly, the sample of NO_3_^−^ medium, for determining the presence of bacteria (Section 2.5), was also filtered in the same manner. Analysis of NO_3_^−^ concentrations was performed using the Griess Reagent system as previously described by Miranda et al. [47]. Blood samples were analysed at baseline, week 2 (WK2) and week 4 (WK4), whilst medium samples were only analysed at baseline. Medium samples were diluted accordingly to ensure the NO_3_^−^ values would fall within the standard range. Sodium nitrate was used for standard quantification and the results were expressed in micromoles per litre (µM). The coefficients of variation (*n* = 35, in duplicate) of the methods were 2.9% to intra-assay and 4.0% to inter-assay.

### 2.9. Statistical Analysis

Per-protocol analysis was utilized for the present study. The assumption of normality, homogeneity of variances and sphericity were examined with the Shapiro–Wilk, Levene’s and Mauchly tests, respectively. For the primary and secondary outcomes, the change in each parameter was compared between placebo and dietary nitrate arms by an unpaired Student *t*-test. Changes in parameters over time were compared by repeated measures analysis of variance (ANOVA) with Bonferroni post hoc tests (to account for multiple comparisons) and unpaired Student *t*-test for comparisons between groups. The level for statistical significance taken was *p* < 0.05 for all analyses. If groups were different at baseline for any of the dependent variables, an analysis of covariance (ANCOVA) was used to test for differences between groups at two weeks and four weeks with baseline values being included as the covariate. Results for all normally distributed data are presented as mean ± standard deviation (SD). Data that were skewed were log transformed, however the median and interquartile range (IQR) of the raw data are presented. All analyses were performed using a commercially available statistical package (IBM SPSS Statistics version 22 for Windows, Chicago, IL, USA).

## 3. Results

A total of 18 participants completed all three study visits between January 2017 to December 2018. According to verbal confirmation, both interventions were well tolerated for the duration of the study. No serious adverse events were experienced, however common side effect such as beeturia and faecal discolouration were reported. Baseline demographic characteristics were similar for both treatment allocations (Table 1). None of the participants had any uncontrolled conditions and were considered to be healthy individuals. 

### 3.1. Plasma Nitrate Concentrations 

**Figure 2:**
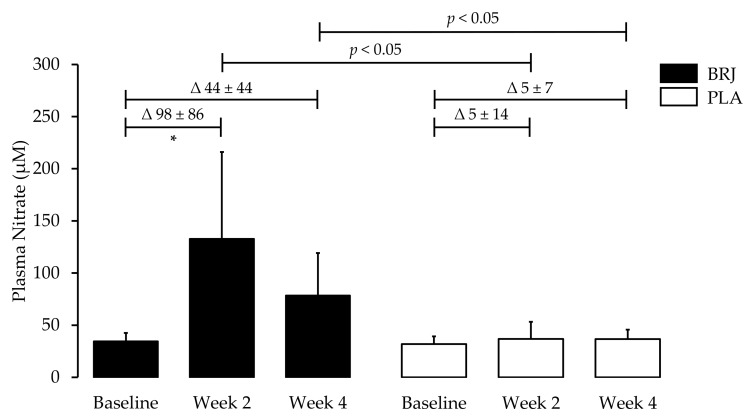
The effects of four weeks of BRJ consumption or PLA on plasma nitrate. Beetroot juice consumption increases plasma nitrate in healthy older adults. Data are expressed as mean ± SD. * Significance shown for comparisons within treatment allocations of the change between baseline, week 2 and week 4, *p* < 0.05 for Bonferroni post hoc test. Significance shown for comparisons between treatment allocations for an unpaired Student *t*-test, *p* < 0.05. BRJ = beetroot juice; PLA = placebo.

Plasma samples were analysed for 12 individuals (6 BRJ; 6 PLA), due to insufficient volumes from the remaining six participants (Figure 2). Across the duration of the study, NO_3_^−^ levels were highest in the BRJ group following two weeks of supplementation but declined slightly from WK2 to WK4. Mean plasma NO_3_^−^ concentrations of the BRJ group increased from 34 ± 8 µM to 132 ± 83 µM following two weeks (*p* = 0.04), with concentrations measuring at 78 ± 41 µM at four weeks (*p* = 0.60). Furthermore, NO_3_^−^ levels were significantly greater compared to PLA treatment at WK2 and WK4 (*p* = 0.02; *p* = 0.03, respectively). There were no significant changes in plasma NO_3_^−^ concentration within the PLA group.

### 3.2. Resting Blood Pressure 

**Figure 3:**
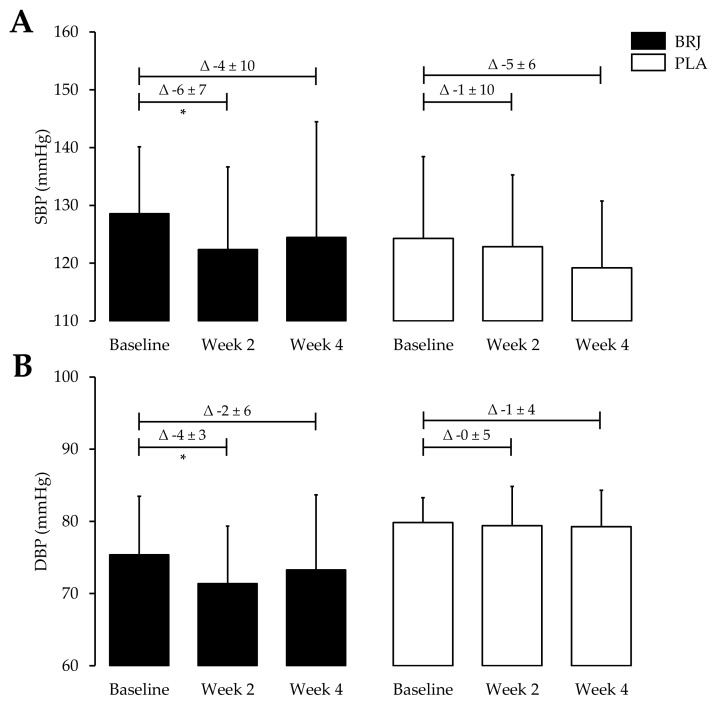
The effects of four weeks of BRJ consumption or PLA on BP. Beetroot juice consumption reduces (**A**) SBP and (**B**) DBP in healthy older adults. Data are expressed as mean ± SD. * Significance shown for comparisons within treatment allocations of the change between baseline, week 2 and week 4, *p* < 0.05 for a Bonferroni post hoc test. BRJ = beetroot juice; DBP = diastolic blood pressure; PLA = placebo and SBP = systolic blood pressure.

Following two weeks of BRJ ingestion, SBP reduced by −6 ± 7 mmHg and DBP by −4 ± 3 mmHg (*p* = 0.04; *p* = 0.01, respectively). However, from WK2 to WK4, SBP increased by 2 ± 7 mmHg (*p* = 1.00) and DBP by 2 ± 4 mmHg (*p* = 0.49). Over the four-week course of the BRJ treatment, SBP reduced by −4 ± 10 mmHg (*p* = 0.67) and DBP by −2 ± 6 mmHg (*p* = 0.75). Within the PLA treatment group, SBP reduced by −1 ± 10 mmHg following two weeks and DBP by 0 ± 5 (*p* = 1.00). From WK2 to WK4, SBP was further reduced by −4 ± 7 mmHg (*p* = 0.70) and DBP by 0 ± 3 mmHg (*p* = 1.00). Over the four-week course of the PLA treatment, SBP reduced by −5 ± 6 mmHg (*p* = 0.17) and DBP by −1 ± 4 mmHg (*p* = 1.00). None of the BP changes for PLA treatment reached statistical significance. Additionally, no significant differences were observed between the groups at any time point (Figure 3). Similarly, the changes in BP were not significant between the two groups after two and four weeks (Table 2).

### 3.3. Dietary NO_3_^−^ and NO_2_^−^ Intake

Analysis of the three-day self-report food diary was performed in order to quantify the NO_3_^−^ and NO_2_^−^ intake at baseline, before their second visitation and before their final visitation (Table 3). At each time-point, the intake of NO_3_^−^ and NO_2_^−^ did not significantly differ between groups. Furthermore, no difference was observed over the four weeks within the BRJ and PLA groups.

### 3.4. Presence of NO_3_^−^ Reducing Bacteria

Twelve participants were included for the oral microbiome assessment (6 BRJ; 6 PLA). This assessment was used to determine the presence of NO_3_^−^ reducing bacteria. Every participant who was included for this assessment demonstrated the capacity to reduce NO_3_^−^. Following 54 h in incubation, the tongue scrape samples from each participant successfully reduced all of the NO_3_^−^ in the broth medium, meaning a 100% reduction of the available NO_3_^−^ substrate. These results confirmed the presence of NO_3_^−^ reducing bacteria in the oral cavity of the 12 participants included for this analysis.

### 3.5. Microvascular Function

**Figure 4:**
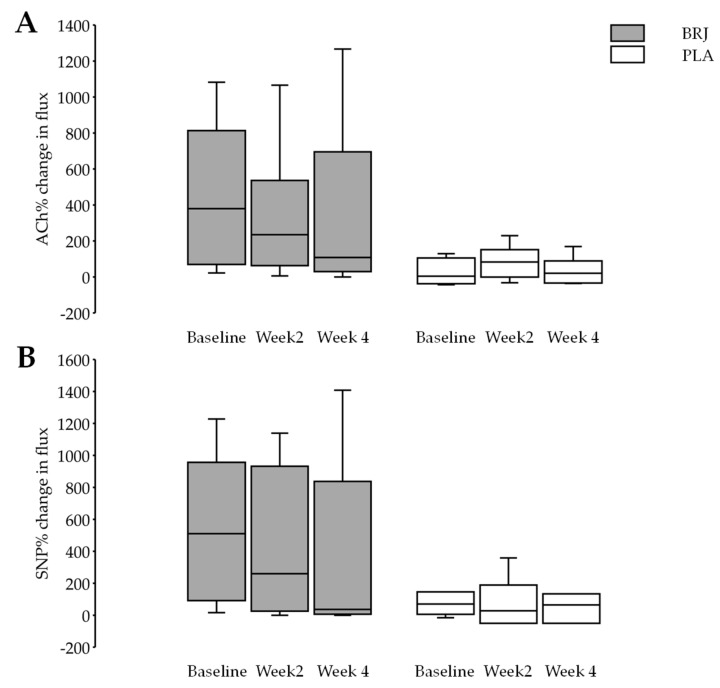
The effect of treatments on laser Doppler imaging (LDI) with iontophoresis is over four weeks (*n* = 18). Log-transformed data was used for all analyses, as the untransformed data was not normally distributed. Untransformed data expressed as median (IQR). ACh = acetylcholine; LDI = laser Doppler imaging; IQR = interquartile range and SNP = sodium nitroprusside.

Blood flux was quantified by calculating the median rather than the mean for each region of interest. This is due to the fact the laser Doppler imaging data will rarely have Gaussian distribution [48]. The flux responses to ACh and SNP were not normally distributed for any of the laboratory visits, therefore log-transformed data was used to test for differences. However, the results are presented using raw data as percentage changes in flux with IQR (Figure 4). Contrary to our hypothesis, no significant differences in endothelium-dependent (ACh) or endothelium-independent (SNP) microvascular responses were observed over the three visitations.

As expected, flux values increased in response to the iontophoresis of ACh for both groups. During the baseline visit, the median responses were measured at 380.0% (IQR: 743.6%) for the BRJ group and 44.3% (IQR: 133.3) for the PLA (*p* = 0.03). Significant differences between groups were reported at baseline; therefore, these values were used as a covariate in ANCOVA analysis to test for difference between groups at WK2 and WK4. Median responses at WK2 were 234.9% (IQR: 473.8%) for BRJ group and 117.8% (IQR: 141.8%) for the PLA group (*p* = 0.75). Furthermore, no significant differences between groups was observed at WK4, with the BRJ response measured at 108.1% (IQR: 665.45%) and PLA response measured at 59.1% (IQR: 130.94%; *p* = 0.42). Similarly, the changes in ACh were not significant between the two groups after two and four weeks (Table 2).

As expected, flux values increased in response to the iontophoresis of SNP for both groups. During the baseline visit, the median responses were measured at 510.0% (IQR: 864.5%) for the BRJ group and 112.3% (IQR: 131.0) for the PLA (*p* = 0.15). Median responses at WK2 were 260% (IQR: 906.4%) for BRJ group and 73.1% (IQR: 223.3%) for the PLA group (*p* = 0.43). Furthermore, no significant differences between groups was observed at WK4, with the BRJ response measured at 33.4% (IQR: 830.8%) and PLA response measured at 107.0% (IQR: 171.9%; *p* = 0.80). Similarly, the changes in SNP were not significant between the two groups after two and four weeks (Table 2).

### 3.6. Large-Vessel Endothelial Function 

**Figure 5:**
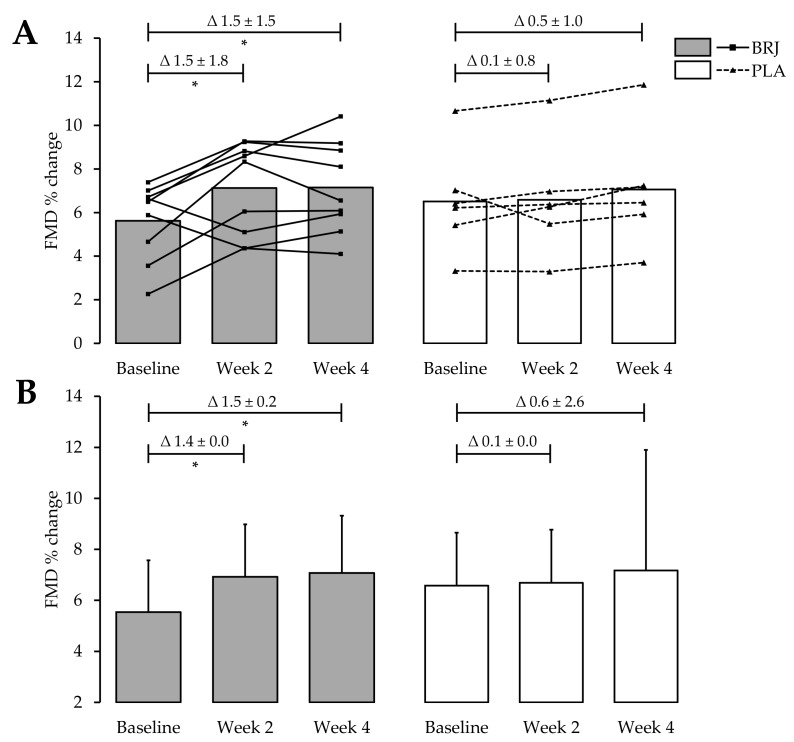
The effects of four weeks of BRJ consumption or PLA on FMD. Beetroot juice consumption increases FMD responses in healthy older adults. Both (**A**) uncorrected and (**B**) allometrically scaled data are expressed as mean ± SD and individual data for uncorrected data. * Significance shown for comparisons within treatment allocations of the change between baseline, week 2 and week 4, *p* < 0.05 for the Bonferroni post hoc test. BRJ = beetroot juice; FMD = flow-mediated dilatation and PLA = placebo.

Data loss occurred for three participants, therefore only 15 participants (9 BRJ; 6 PLA) were included for the main FMD analysis. FMD responses increased from baseline to WK2 for the BRJ treatment and the improvements were sustained until WK4. At baseline, endothelium-dependent vasodilation was 5.6% ± 1.8% in the BRJ group and for the PLA group it was 6.4% ± 2.5% (*p* = 0.49). Within the BRJ group, FMD values increased by 1.5% ± 1.8% at WK2 (*p* = 0.04), however values only increased by 0.1% ± 0.8% in the PLA groups (*p* = 0.84). After four weeks, FMD values increased by 1.5% ± 1.5% for the BRJ treatment (*p* = 0.01). The PLA group failed to reach any significance after four weeks, although a small improvement of 0.5% ± 1.0% was reported (*p* = 0.23). Further to this, no main effect between groups was found at WK2 (*p* = 0.58) or WK4 (*p* = 0.85; Figure 5A). Similarly, the changes in FMD were not significant between the two groups after two and four weeks (Table 2). Using appropriate allometric scaling methods on the data did not change any of the outcomes; however, corrected data is presented in Figure 5B. 

## 4. Discussion

The findings from the present pilot study indicate that medium-term BRJ supplementation could potentially improve large-vessel EF and BP in healthy older adults. Despite our findings showing no main effect between groups, significant improvements in both SBP and DBP were reported within the BRJ group after two weeks. However, the changes in BP were not significantly different to BP changes in the PLA group. After four weeks, SBP and DBP values remained lower than baseline within the BRJ group, but were no longer significant. Interestingly, a large reduction of −5 ± 6 mmHg in SBP was observed in the PLA group following four weeks and could potentially mask any between group effects. Although the exact cause of these improvements was unknown, we concluded that they could be due to the increase in IPAQ scores, which were not observed in the BRJ group. Alternatively, it has been proposed that on average systolic and diastolic BP reduce by 15 mmHg and 7 mmHg respectively by the third visit with a physician [49]. However, given that both groups would likely experience this effect, the magnitude of BP change in BRJ is likely to be mediated by the increased NO_3_^−^ levels from the BRJ. Contrary to our hypothesis, the present pilot study observed no changes in microvascular function in response to the intervention. This null finding cannot be explained by non-responders as we demonstrated that participants had elevated plasma NO_3_^−^ levels and a full capacity to convert the NO_3_^−^ within the oral microbiome. 

A vast amount of research has demonstrated the significance of nutritional therapy for cardiovascular health, particularly for high BP [43,44]. In recent years, dietary NO_3_^−^ is one nutraceutical that has been identified as a treatment for hypertension (average reductions: SBP; −4.4 mmHg and DBP; −1.1 mmHg) [50]. Aside from the present pilot study, only a few have tested medium-term interventions on EF and BP in humans (≥4 weeks) [18,19]. According to a comprehensive review by Carlström et al., this therapeutic effect is achieved through several mechanisms [51]. The evidence implies that improvements in EF were the primary explanation for these reductions. Indeed, exogenous sources of NO have been shown to induce vasodilation, which consequently reduces SVR [52]. Interestingly, these vascular responses to dietary NO_3_^−^ consumption occur shortly after ingestion and coincide with BP reductions. In support of this statement, Bondonno et al. demonstrated significant improvements in FMD responses 2 h post NO_3_^−^ ingestion, which were also accompanied by reductions in SBP.

To date, the longest intervention performed in humans is a six-week study, in hypercholesterolemic individuals [18]. The authors reported that over the duration of the study, FMD responses improved by 4% and both SBP and DBP were reduced (−4.1 mmHg and −1.5 mmHg, respectively). Further support for medium-term supplementation was published by Kapil et al. in 2015. They reported a 20% improvement in FMD responses following four weeks of BRJ supplementation in a hypertensive cohort, which was accompanied by reductions in SBP and DBP (−7.7 mmHg and −2.4 mmHg, respectively) [19]. Similarly, the present pilot study found improvements of 27% in FMD responses and reductions of −4.1 mmHg and −2.1 mmHg in SBP and DBP, respectively. Despite our pilot data not showing a significant effect between the groups, collectively, these positive findings imply that medium-term use of NO_3_^−^ supplements can reduce BP and improve EF of the large blood vessels. However, despite the microvasculature being the primary regulator of SVR [53], only a few studies have investigated the short or medium-term use of dietary NO_3_^−^ on microvascular function. 

Contrary to our hypothesis, four weeks of NO_3_^−^ supplementation did not lead to significant changes in endothelium-dependent or endothelium-independent microvascular reactivity. A potential reason for this null finding could be explained by the pharmacologically vasoactive factors that were administered. Neither, ACh or SNP stimulates the conversion of NO_2_^−^ into NO, similar to what happens under the ischemic conditions of the FMD procedure. However, Wong et al. stimulated microvascular vasodilation using reactive hyperaemia and found that three days of BRJ supplementation did not alter cutaneous vascular reactivity [54]. The authors proposed that cutaneous reactive hyperaemia is not NO mediated and is rather to be largely dependent on cutaneous sensory-nerve and/or calcium activated potassium (BKCa) channels [55]. It should be noted that this group used laser Doppler flowmetry to measure cutaneous blood flux, which only measures perfusion at a single point. Importantly, laser Doppler imaging scans a larger region of interest and thereby accounts for the spatial heterogeneity of skin blood flow [56]. Therefore, further research is warranted in order to investigate the effects of NO_3_^−^ supplements on the microcirculation.

The participants for the present pilot study were recruited from a healthy aging population and most of them showed no signs of hypertension. Although significant improvements in large-vessel EF and BP were observed after two weeks within the BRJ group, it was not expected for improvements to continue indefinitely due to the good health of the participants. However, the changes in plasma NO_3_^−^ levels corresponded with the changes in BP, thus when plasma NO_3_^−^ levels decreased from two weeks to four weeks, BP increased. Therefore, an alternative reason why improvements were not sustained for the duration of the study could be explained by the reductions in circulating NO_3_^−^. The exact cause of the diminished NO_3_^−^ availability is not known. Some authors believe that NO_3_^−^ and NO_2_^−^ clearance is upregulated with prolonged exposure [57], however, compliance to this intervention was only confirmed verbally, therefore we are unsure if every participant adhered to their allocated drinks.

### Limitations

A limitation of the present pilot study was the small sample size, due to difficulty with recruitment. A-priori sample size calculation was not performed and therefore we are unable to confirm whether the findings are adequately powered and the result should be interpreted with caution. Additionally, as previously mentioned, compliance to the intervention was only confirmed verbally at the end of the trial; but biological markers did support this declaration to an extent. Although plasma NO_3_^−^ levels were measured, we did not have the facilities to analyse NO_2_^−^ concentrations. Previous finding suggests that significant increases in plasma NO_2_^−^ occur alongside increases in plasma NO_3_^−^ in elderly people with T2D [30]. Unfortunately, we were unable to analyse plasma NO_3_^−^ nor NO_3_^−^ reducing bacteria for all our participants, but the available data suggests that we did not recruit non-responders. Furthermore, it should be noted that the 12 participants who were included for these assessments were not necessarily the same individuals. The large variation in microvascular flux between groups at baseline was also a limitation of the present study. While we accounted for this difference during analysis of the follow-up visits, we are unclear about the exact reason for this difference. 

## 5. Conclusions

In conclusion, this pilot study demonstrated that medium-term BRJ ingestion potentially improves SBP, DBP and large-vessel EF in healthy older adults. These changes corresponded to the changes in plasma NO_3_^−^ levels. Although no changes in microvascular function were observed, further prospective studies examining the long-term impact of NO_3_^−^ supplementation on this vascular bed in patient populations at risk of CVD are warranted. 

## Figures and Tables

**Table 1:** Baseline characteristics stratified by treatment allocation.

Treatment Allocation	BRJ	PLA	Significance
Demographics			
*n*	11	7	
Age (y)	65 ± 8	61 ± 5	0.34
Height (cm)	167.4 ± 9.5	165.3 ± 6.2	0.62
Weight (kg)	73.2 ± 16.6	73.3 ± 8.0	0.99
BMI (kg/m^2^)	26.2 ± 6.3	26.9 ± 2.1	0.80
Body Fat (%)	30.7 ± 10.5	33.8 ± 7.7	0.51
Dietary Intake			
NO_3_^−^	151.8 ± 77.8	185.3 ± 75.7	0.78
NO_2_^−^	7.4 ± 3.8	5.8 ± 1.9	0.49
Clinical BP			
SBP	129 ± 12	124 ± 14	0.46
DBP	75 ± 8	79 ± 3	0.23

Data are presented as mean ± SD. *p*-values shown in the last column for an unpaired Student *t*-test. BMI = body mass index; BRJ = beetroot juice; DBP = diastolic blood pressure; NO_3_^−^ = nitrate; NO_2_^−^ = nitrite; PLA = placebo and SBP = systolic blood pressure.

**Table 2:** Changes in vascular outcomes during the intervention.

	BRJ						PLA						P (Comparison between BRJ and PLA)		
	Baseline	Week 2	Week 4	∆ _Week 2 − Baseline_	∆ _Week 4 − Week 2_	∆ _Week 4 − Baseline_	Baseline	Week 2	Week 4	∆ _Week 2 − Baseline_	∆ _Week 4 − Week 2_	∆ _Week 4 − Baseline_	∆ _Week 2 − Baseline_	∆ _Week 4 − Week 2_	∆ _Week 4 − Baseline_
SBP (mmHg)	129 ± 12	122 ± 14	124 ± 20	−6 ± 7	2 ± 7	−4 ± 10	124 ± 14	123 ± 12	119 ± 11	−1 ± 10	−4 ± 7	−5 ± 6	0.23	0.12	0.84
DBP (mmHg)	75 ± 8	71 ± 8	73 ± 10	−4 ± 3	2 ± 4	−2 ± 6	79 ± 3	79 ± 5	79 ± 5	0 ± 5	0 ± 3	−1 ± 4	0.08	0.29	0.55
ACh (%)	447.30 ± 364.82	304.59 ± 326.87	343.65 ± 425.80	−142.71 ± 295.97	39.05 ± 411.12	−103.66 ± 340.20	66.16 ± 65.02	116.30 ± 88.28	68.58 ± 71.58	50.14 ± 142.28	−47.72 ± 143.64	2.42 ± 71.20	0.13	0.60	0.43
SNP (%)	537.53 ± 434.81	449.00 ± 451.84	358.91 ± 534.79	−88.54 ± 341.96	−90.09 ± 497.00	−178.62 ± 357.99	216.99 ± 305.40	122.15 ± 143.08	171.28 ± 279.98	−98.84 ± 373.01	49.13 ± 364.91	−45.71 ± 394.21	0.97	0.53	0.47
FMD (%)	5.26 ± 1.75	7.12 ± 2.12	7.15 ± 2.09	1.50 ± 1.81	0.02 ± 1.03	1.53 ± 1.49	6.38 ± 2.36	6.45 ± 2.52	6.91 ± 2.63	0.07 ± 0.83	0.46 ± 0.33	0.54 ± 0.97	0.10	0.34	0.18

Data are presented as mean ± SD. An unpaired Student *t*-test was used to evaluate differences in changes (∆) between the two groups. ACh = acetylcholine; BRJ = beetroot juice; DBP = diastolic blood pressure; FMD = flow-mediated dilatation; PLA = placebo; SBP = systolic blood pressure and SNP = sodium nitroprusside.

**Table 3:** Quantification of the amount of NO_3_^−^ and NO_2_^−^, measured with a three-day self-report food diary.

Treatment Allocation	BRJ	PLA	Significance
NO_3_^−^ (mg)			
Baseline	151.8 ± 77.8	185.3 ± 75.7	1.00
WK2	182.5 ± 72.4	200.2 ± 63.2	0.66
WK4	195.4 ± 66.9	190.4 ± 113.6	0.47
NO_2_^−^ (mg)			
Baseline	7.4 ± 3.8	5.8 ± 1.9	0.12
WK2	6.2 ± 4.0	8.2 ± 2.9	0.43
WK4	8.1 ± 4.4	5.1 ± 2.3	0.07

Data are presented as mean ± SD. *p*-values shown in the last column for an unpaired Student *t*-test. BRJ = beetroot juice; NO_3_^−^ = nitrate; NO_2_^−^ = nitrite; PLA = placebo; WK2 = week 2 and WK4 = week 4.
